# Supplementary material for: Modulation of the Gut Microbiota by Tomato Flours Obtained after Conventional and Ohmic Heating Extraction and Its Prebiotic Properties
Source: Foods. 2023 May 8;12(9):1920. doi: 10.3390/foods12091920 (PMC10178612; doi:10.3390/foods12091920)
Supplement: Supplementary file 1 [file foods-12-01920-s001.zip › foods-2249671-supplementary.pdf]

Suplementary material

Table S1. Real time PCR conditions.

PCR stage	Temperature (ºC)	Time (s)	Number of cycles
Initial denaturation/ enzyme activation	95	600	45
Denaturation	95	10	
Annealing	Specific temperature for each primer	60	
Extension*	72	15	
Melting curve*	60-97, with an increment of 0.5 for 0.05 min	0.05	

*Stages in which fluorescence is measured

Table S2. Primer sequences targeting bacterial groups, genomic DNA standards and PCR product size.

	Primer	Sequence	Genomic DNA standard	PCR Product size	Annealing temperatures (ºC)
**Firmicutes**	Forward	ATG TGG TTT AAT TCG AAG CA	*Lactobacillus gasseri*	126	45
	Reverse	AGC TGA CGA CAA CCA TGC AC	ATCC 33323		
**Bacteroidetes**	Forward	CAT GTG GTT TAA TTC GAT GAT	*Bacteroides vulgatus*	126	45
	Reverse	AGC TGA CGA CAA CCA TGC AG	ATCC 8482		
**Bacteroides**	Forward	ATA GCC TTT CGA AAG RAA GAT	*Bacteroides vulgatus*	495	45
	Reverse	CCA GTA TCA ACT GCA ATT TTA	ATCC 8482		
Clostridium leptum subgroupIV	Forward	GCA CAA GCA GTG GAG T	*Clostridium leptum*	239	45
	Reverse	CTT CCT CCG TTT TGT CAA			
**Lactobacillus**	Forward	GAG GCA GCA GTA GGG AAT CTT C	Lactobacillus gasseri	126	55
	Reverse	GGC CAG TTA CTA CCT CTA TCC TTC TTC	ATCC 33323		

**Bifidobacterium**	Forward	CGC GTC YGG TGT GAA AG	*Bifidobacterium longum subsp. Infantis*	244	50
	Reverse	CCC CAC ATC CAG CAT CCA	ATCC 15697		
**Roseburia**	Forward	AGGCGGTACGGCAAGTCT	Roseburia intestinalis DSM14610		56
	Reverse	AGGCGGTACGGCAAGTCT			
**Prevotella**	Forward	CACCAAGGCGACGATCA	Prevotella	283	56
	Reverse	GGATAACGCCCGGACCT			

Table S3. The proximate composition and total phenolic compounds of SFOH and SFCONV samples (g/100g).

**Chemical Composition** (g/100g DW)			**Sample (g/100 g)**	
			**SFOH**	**SFCONV**
Proximate composition		*Ash*	3.32 ± 0.21	2.98 ± 0.16
		Protein	18.72 ± 0.47*	16.29 ± 0.59
		Total Fatty acids	21.12 ± 0.51*	17.82 ± 0.3
		Dietary Fiber		
		TDF	62.47 ±1.24*	59.06 ± 0.67
		IDF	50.99 ± 0.16*	46.01 ± 0.13
		SDF	10.86 ± 0.85*	12.98 ± 0.64
Phenolic composition	***Free phenolic extract (FPC)***	*TPC*	1.46 ± 0.05*	0.8 ± 0.01
		ABTS	0.28 ± 0.01	0.99 ± 0.15*
		ORAC	2.44 ± 0.34*	1.6 ± 0.09
	Bound phenolic extract (BPC)	*TPC*	0.42 ± 0.03	0.73 ± 0.12*
		ABTS	2.23 ± 0.21	2.63 ± 0.11
		ORAC	2.41 ± 0.22	2.71 ± 0.43

Values are the mean of three replicates of three independent experiments ± standard deviation. TDF- total dietary fiber; IDF; insoluble dietary fiber; SDF- soluble dietary fiber; TPC- total phenolic compounds (g galllic acid Eq./100g DW); ABTS Antioxidant activity by ABTS method (g ascorbicacid eq. / 100 gDW); ORAC- antioxidant activity (g trolox eq./ 100g DW).Values marked by the asterisk within the same column are statistically significantly different (determined by ANOVA, $p < 0.05$).

Table S4. Constituents (g/100 g fibre DW) of SDF and IDF from SFOH and SFCONV samples.

	**SFOH**		**SFCONV**	
	SDF	IDF	SDF	IDF
**Klason Lignin**		13.06 ± 0.52 [b]		14.09 ± 0.27[a]
**Glucose (as cellulose)**	24.42 ± 0.43[a]	55.32 ± 1.21[b]	32.1 ± 0.56[c]	50.23 ± 1.76[d]

**Hemicellulose**	15.12 ± 0.54 ^{a}	25.2 ± 0.10 ^{b}	12.71 ± 1.21 ^{a}	24.72 ± 0.30 ^{b}
**Xylose**	*	13.2 ± 0.10 ^{a}	*	15.1 ± 0.17^{b}
**Galactose**	1.87 ± 0.21	*	*	0.31 ± 0.021
**Manose**	*	*	4.10 ± 0.12 ^{a}	*
**Arabinose**	13.25 ± 0.86	12.81 ± 0.28	12.71 ± 1.21	9.31 ± 0.51
**Uronic acids**^{*1}	67.41 ± 2.15 ^{a}	81.92 ± 1.98 ^{b}	58.93 ± 2.56 ^{c}	78.49 ± 2.32 ^{b}
**Resistant Protein**		16.03 ± 0.05 ^{a}		11.69 ± 0.03 ^{b}
**Bond Phenolic compounds**^{*2}	7.80 ± 0.43 ^{a}	31.18 ± 2.31 ^{b}	4.99 ± 0.35 ^{c}	34.10 ± 1.21 ^{b}

* < LOD. TDT - Total dietary fibre; IDF – insoluble dietary fibre; SDF – Soluble dietary fibre. ^{*1} - mg GUAE / g fibre dry weight; *2 - mg GAE/ 100g fibre DW. Results are the means of three determinations ± standard deviation. Different letters in the same column are significantly different, as determined by ANOVA ($p< 0.05$).

Table S5. Recovery index and bioaccessibility of total phenolic compounds, antioxidant activity, and individual phenolic compounds from SFCONV and SFOH samples throughout digestion.

**Bioactivities**	**Samples**	**Mouth**	**Colon**
Total Phenol	SFCONV	101.42 ± 2.34^{a}	90.78 ± 1.12 ^{d}
	SFOH	70.44 ± 1.56 ^{a}	103.04 ± 1.28 ^{d}
ABTS	SFCONV	103.08 ± 2.23 ^{a}	98.49 ± 2.45 ^{a}
	SFOH	81.45 ± 1.87 ^{a}	92.51 ± 1.96
Orac (trolox eq.)	SFCONV	109.34 ± 2.25 ^{a}	90.49 ± 2.54
	SFOH	89.19 ± 2.54 ^{a}	87.50 ± 1.26
gallic acid	SFCONV	101.19 ± 1.87 ^{a}	n.d.
	SFOH	134.31 ± 1.12	n.d.
4-hydroxybenzoic acid	SFCONV	100.38 ± 1.56 ^{a}	n.d.
	SFOH	102.54 ± 1.69 ^{a}	70.98 ± 0.13 ^{c}
p-coumaric acid	SFCONV	79.35 ± 2.56 ^{a}	36.15 ± 0.89 ^{d}

	SFOH	89.07 ± 1.98 [a]	0.42 ± 0.03 [d]
rutin	SFCONV	17.01 ± 0.54 [a]	n.d.
	SFOH	7.84 ± 0.05 [a]	n.d.

n.d. not determined. Recovery index and bioaccessibility %. Results are the means of three determinations ± standard deviation. Different letters in the same line are significantly different ($p<0.05$), the greek alphabet means significant differences between methods used in the same column ($p<0.05$). as determined by ANOVA ($p< 0.05$).

Table S6. Recover index and bioaccessibility percent of carotenoids identified by mass spectrometry LC-ESI-UHR-QqTOF-MS to OH and extracts.

	**Mz**	**Mouth (%)**		**Colon (%)**	
Samples		OH	CONV	OH	CONV
n.i	525	84.85 ± 2.31	86.32 ± 2.05	40.33 ± 1.87	41.32 ± 1.98
n.i	527	91.55 ± 1.95	93.41 ± 1.28	92.48 ± 2.06	90.23 ± 2.77
phytofluene	542	72.25 ± 1.04	26.40 ± 0.27	69.82 ± 1.76	35.54 ± 1.43
lycopene	536	n.q	n.q	n.q	n.q
lutein	569	113.94 ± 2.76	25.53 ± 0.34	19.26 ± 1.25	30.53 ± 1.23
n.i.	633	103.78 ± 2.54	n.d	12.98 ± 0.12	n.d

n.i. non identified; n.d. non determinate; n.i. non identified .

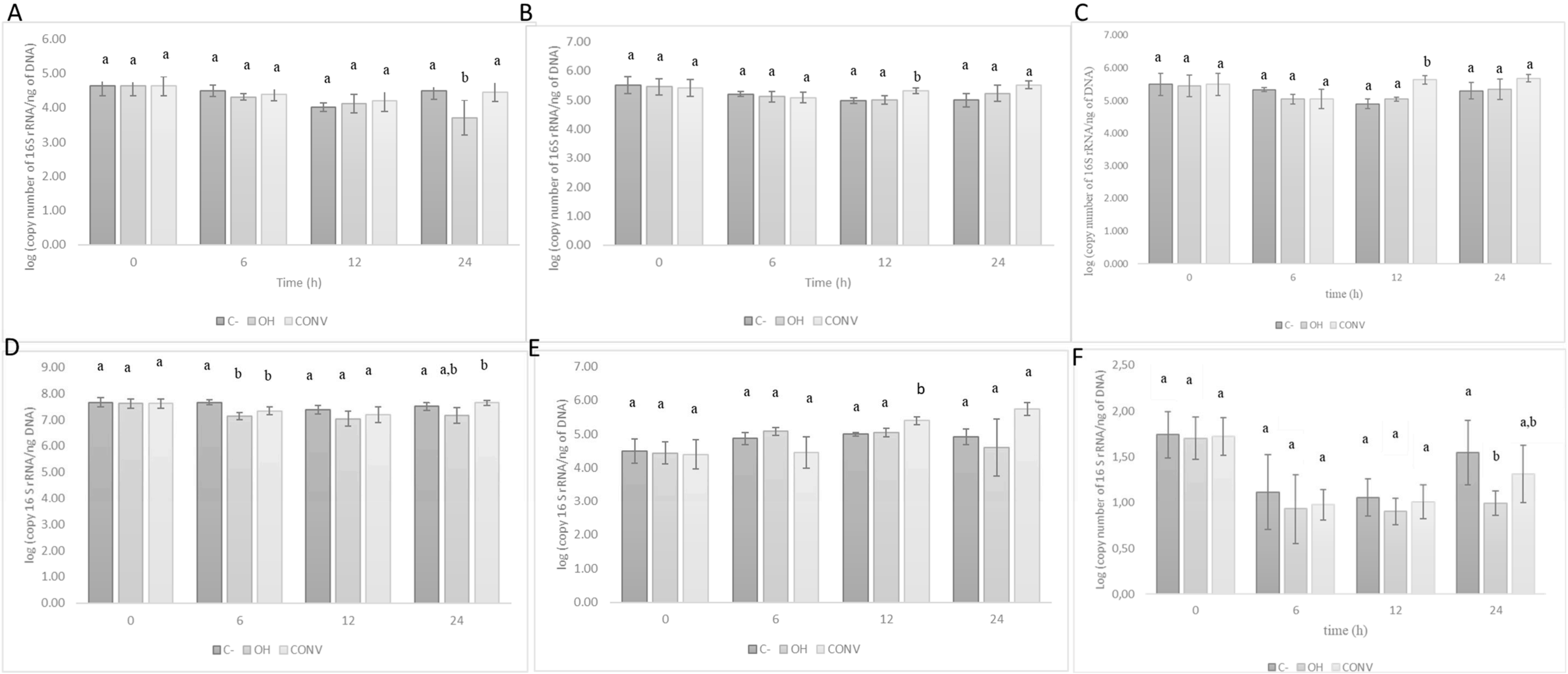

Figure S1. Bacterial populations (log (copy number of 16 S rRNA/ng of DNA), means ± SD) detected by PCR in Fecal samples. The used probes: Clostridium leptum (A), Bacteroidetes (B), Bacteroides (C), Firmicutes (D), Bifidobacterium (E), and Akkermansia (F) Different letters mark statistically significant ($p<0.05$) differences between samples at each sampling point.
